# Effects of Flash Glucose Monitoring on Dietary Variety, Physical Activity, and Self-Care Behaviors in Patients with Diabetes

**DOI:** 10.1155/2020/9463648

**Published:** 2020-04-09

**Authors:** Satoshi Ida, Ryutaro Kaneko, Kanako Imataka, Kaoru Okubo, Yoshitaka Shirakura, Kentaro Azuma, Ryoko Hujiwara, Hiroka Takahashi, Kazuya Murata

**Affiliations:** Department of Diabetes and Metabolism, Ise Red Cross Hospital, Ise-shi, Mie 516-8512, Japan

## Abstract

The aim of this study was to evaluate the effects of flash glucose monitoring on dietary variety, physical activity, and self-care behavior in patients with diabetes. This study included outpatients with diabetes using insulin who presented at the Department of Diabetes and Metabolism of the Ise Red Cross Hospital. Before initiating flash glucose monitoring and 12 weeks after its initiation, blood glucose-related parameters were assessed and self-administered questionnaires were completed (Dietary Variety Score (DVS), the International Physical Activity Questionnaire (IPAQ), the Summary of Diabetes Self-Care Activities Measure (SDSCA), and the Diabetes Treatment Satisfaction Questionnaire (DTSQ)) and compared between the two time points. We analyzed 42 patients with type 1 diabetes mellitus and 48 patients with type 2 diabetes mellitus. In patients with type 2 diabetes mellitus, but not type 1 diabetes mellitus, there was an increase in moderate/high category scores for IPAQ (*P* < 0.001) and for treatment satisfaction reported via DTSQ. Furthermore, in patients with type 2 diabetes mellitus, the glycemic excursion index improved significantly and HbA1c decreased significantly (from 7.7 (1.2) to 7.4 (0.8), *P* = 0.025). Results showed that standard deviation and mean amplitude of glycemic excursions significantly decreased in patients with type 1 diabetes mellitus (from 71.2 (20.4) to 66.2 (17.5), *P* = 0.033 and from 124.6 (31.9) to 108.1 (28.4), *P* < 0.001, respectively). Flash glucose monitoring is a useful tool to improve physical activity in patients with type 2 diabetes.

## 1. Introduction

The number of patients with diabetes worldwide is increasing and is estimated to reach 300 million by 2050 [1, 2]. Strict glycemic control is important to minimize the onset of microvascular disorders and microangiopathy [3, 4]. The standard treatment regimen for patients with diabetes includes diet and exercise therapies. When glucose levels no longer respond to such regimens, drug therapy is initiated. However, in real clinical scenarios, these treatment interventions alone fail to achieve good glycemic control in many patients [5, 6]. Frequent use of insulin is required not only in patients with type 1 diabetes mellitus (T1DM) but also in those with type 2 diabetes mellitus (T2DM) because of poor glycemic control. This frequent treatment poses an increased risk of hyperglycemia and hypoglycemia, thereby resulting in a high glucose variability [7, 8], which is closely associated with an increased risk of complications, such as cardiovascular diseases [9], mortality [10], and a decreased quality of life (QOL) [11]. Thus, good-quality glycemic control in patients with diabetes is essential. Improvement of the mean glucose levels, along with attenuation of glucose variability, is clinically very important for patients with diabetes who use insulin.

Flash glucose monitoring (FGM), which is a sensor-based glucose monitoring system, allows patients to record their subcutaneous interstitial fluid glucose level by receiving data from a sensor placed on the upper arm for up to 14 days [12]. The recorded glucose level can be read by scanning with a dedicated reader. Previous studies reported that the use of FGM decreased the duration of hypoglycemia and glucose variability in patients with T1DM [13] and T2DM who use insulin [14]. Another study reported improvement of QOL and treatment satisfaction with FGM use [15]. Therefore, FGM is a device that can improve glucose-related parameters and QOL.

However, such improvement mechanisms associated with FGM remain to be elucidated. Some studies have demonstrated that FGM use may promote the consumption of balanced foods, physical activity, and self-care activities in patients with diabetes [16, 17]; however, no study has yet evaluated such a relationship. We hypothesized that self-monitoring of recorded glucose levels using FGM would result in changes in the behavior for dietary variety, physical activity, or self-care activities. Therefore, the aim of this study was to evaluate the effects of FGM use on these aspects in patients with diabetes.

## 2. Materials and Methods

### 2.1. Patients and Setting

This study was approved by the ethics board of the Ise Red Cross Hospital, and all patients provided written informed consent. This study was conducted in accordance with the Helsinki Declaration. This study was registered at the University Hospital Medical Information Network (UMIN) Clinical Trials Registry System (trial ID: UMIN 000033275). We included patients with diabetes aged ≥20 years and <75 years who presented at our hospital between July 2018 and March 2019. The inclusion criteria specified patients with T1DM or T2DM with poor glycemic control (HbA1c ≥ 7% and <10% or fasting blood glucose ≥ 110 mg/dL and <250 mg/dL) despite receiving intensive insulin therapy and those who perform self-monitoring of blood glucose (SMBG) at least three times per day before a meal. The exclusion criteria included the following: (1) patients with a history of acute diabetic complications (e.g., diabetic ketoacidosis), pancreatitis, severe infections, alcohol intoxication, severe mental illnesses, or malignant disease; (2) those who developed serious vascular diseases, such as stroke or myocardial infarction, within 6 months before initiation of the study; (3) those who were pregnant or planned to become pregnant; and (4) those who were deemed unfit to participate by the primary physician.

### 2.2. Introduction of FGM

We provided each outpatient with the FGM system, FreeStyle Libre (Abbott Diabetes Care, Witney, Oxon, UK), which they used for 12 weeks. The primary physician and trained nurses instructed patients in FGM use. We instructed the patients about the following: the FGM sensor should be worn on the upper arm, the blood glucose levels should be checked by the FGM with intervals of less than 8 h as much as possible, and whenever hypoglycemia is suspected, the blood glucose level should be checked by self-monitoring. After initiation of FGM use, patients were repeatedly instructed to follow diet and exercise therapies and to perform SMBG at least three times per day. When concomitant diabetic drugs were reduced, discontinued, or added or FGM use was discontinued without permission from the primary physician, the patients were considered dropout participants. The medication dose was adjusted as necessary based on recommendations by the primary physician if HbA1c remained ≥8% 8 weeks after FGM initiation or for those who developed hypoglycemia. Hypoglycemia was defined as (1) a diabetic symptom that is prolonged but disappears after dextrose intake, glucagon administration, or food intake; and (2) glucose levels of <50 mg/dL in SMBG or laboratory tests on presentation with or without symptoms of hypoglycemia. Patients who developed hypoglycemia were treated with oral glucose and were monitored every 15 min until the symptoms disappeared.

### 2.3. Evaluation by Self-Administered Questionnaires

The Dietary Variety Score (DVS) was used to assess dietary intake [18, 19], and the questionnaire included ten food items: fish, meat, eggs, dairy products, soy products, green and yellow vegetables, seaweed, potatoes, fruits, and oils and fats. For each item, patients could select the following frequency categories: “almost every day (1 point),” “once every 2 days (0 points),” “1–2 times per week (0 points),” and “rarely (0 points).” The total score attainable was 10 points, with high scores indicating higher frequency of food intake. To assess physical activity, we used the Japanese version of the International Physical Activity Questionnaire (IPAQ) [20]. The Japanese IPAQ, a reliable tool that was validated in a previous study of patients with diabetes [21], can calculate the mean physical activity per day for 1 week. Levels of physical activity were categorized into low, moderate, and high according to the scoring rule of IPAQ [22]. To evaluate self-care behavior, we used the Japanese version of the Summary of Diabetes Self-Care Activities Measure (SDSCA) [23], which comprises eight factors of standard diet (two items), special diet (three items), overall diet (five items (standard and special diet)), exercise (two items), glucose level self-testing (two items), compliance (two items), foot care (five items), and tobacco use. SDSCA measures the frequency self-care activities performed over 7 days. Response options range from 0 to 7, with higher scores indicating better adherence. To evaluate treatment satisfaction, we used the Japanese version of the Diabetes Treatment Satisfaction Questionnaire (DTSQ) [24] which comprises two categories of treatment satisfaction (the first factor) and perceived frequency of hyper- and hypoglycemia (the second factor). The DTSQ covers eight items, with higher scores indicating higher treatment satisfaction (items: 1, 4, 5, 6, 7, and 8). In addition, the scores for items 2 and 3 are for awareness of hyperglycemia and hypoglycemia, respectively, with higher scores indicating more problematic conditions.

### 2.4. Other Variables

We investigated age, sex, body mass index (weight (kg)/height (m^2^)), smoking status, alcohol consumption, type of diabetes (type 1, 2, or other), duration of diabetes, hemoglobin A1c (HbA1c), hypertension, dyslipidemia, diabetic retinopathy, diabetic neuropathy, diabetic nephropathy, cardiovascular diseases, and use of diabetic agents. Diabetes was classified into types 1 and 2, according to the criteria of the Japan Diabetes Society [25]. Serum lipid and plasma glucose levels were measured using the enzyme method and glucose oxidase method, respectively. HbA1c was evaluated using high-performance liquid chromatography and expressed using the National Glycohemoglobin Standardization Program. Systolic and diastolic blood pressures were measured in the office. Hypertension was defined as meeting any of the following criteria: systolic pressure of ≥130 mmHg, diastolic pressure of ≥80 mmHg, or use of antihypertensive agents. Dyslipidemia was defined as meeting any of the following criteria: triglycerides (TG) of ≥150 mg/dL, high-density lipoprotein cholesterol of <40 mg/dL, low-density lipoprotein cholesterol (LDLC) of ≥120 mg/dL (if coronary artery disease coexists, LDLC of ≥100 mg/dL), or use of lipid-lowering agents. The presence of diabetic retinopathy was evaluated by an ophthalmologist. Patients were defined as having diabetic neuropathy if any of the following was observed: a delay of the Achilles tendon reflex, decreased sensitivity of vibration sense in the lateral malleolus, or abnormal results in a nerve conduction study. Patients were defined as having cardiovascular diseases if they had a present or a past history of ischemic heart disease, such as angina pectoris and myocardial infarction, or cerebrovascular disease, such as cerebral infarction.

### 2.5. Outcome

The outcomes included glucose-related parameters (HbA1c, standard deviation (SD), the mean amplitude of glycemic excursions (MAGE), coefficient of variation (CV), the mean of daily difference (MODD), area above the curve (AAC) of glucose levels of ≥70 mg/dL, and area under the curve of glucose levels of ≥180 mg/dL), weight, scores in self-administered questionnaires, and the amount of insulin used per day.

### 2.6. Safety

After FGM initiation, all events that were considered as adverse were subject to reporting. If a causal relationship was suspected, FGM was discontinued and the relevant patient was regarded as a dropout.

### 2.7. Statistical Analysis

Analysis sets consisted of patients who we were able to follow-up 12 weeks after the initiation of the study, excluding dropouts. Statistical analyses were performed for patient characteristics and changes in outcomes before and after FGM use. Continuous variables between the two groups were compared using a *t*-test, and dichotomous variables were compared using a Chi-square test and the McNemar test. A *P* value that was less than 0.05 and was two sided was considered significant. Analyses were performed with the use of STATA version 12.0 (Stata Corporation LP, College Station, TX). This study was approved by the ethics board of the Ise Red Cross Hospital, and all patients provided written informed consent. This study was conducted in accordance with the Helsinki Declaration. In addition, this study was registered at the UMIN Clinical Trials Registry System (trial ID: UMIN 000033275).

## 3. Results

We enrolled 100 patients who met the inclusion criteria. Ten patients dropped out as they discontinued FGM owing to sensor wear-related rash (five patients) and difficulty with wearing the sensor (five patients). We analyzed the remaining 90 patients.  Table 1 shows the characteristics of the analyzed group. The mean age was 57 years, 52% were female, HbA1c was 7.7%, 42 patients had T1DM, and 48 patients had T2DM. Diabetic agents used by patients included biguanides (64% of patients), dipeptidyl peptidase-4 inhibitors (37% of patients), and sodium-glucose cotransporter 2 inhibitors (37% of patients).

Cardiovascular diseases are defined as angina pectoris, myocardial infarction, stroke, and arteriosclerosis obliterans.

Table 2 shows changes in blood glucose-related parameters before and after FGM initiation. No significant changes in HbA1c values were observed in patients with T1DM. However, there was a significant decrease in SD (from 71.2 (20.4) to 66.2 (17.5), *P* = 0.033) and MAGE (from 124.6 (31.9) to 108.1 (28.4), *P* < 0.001) in these patients. In contrast, in patients with T2DM, there was a significant decrease in HbA1c (from 7.7 (1.2) to 7.4 (0.8), *P* = 0.025) in addition to a significant decrease in SD and MAGE. Furthermore, MODD, an index of daily glucose level difference, significantly decreased (from 49.3 (20.7) to 38.9 (16.3), *P* = 0.001) in patients with T2DM. No significant change in body weight was seen in patients with T2DM; however, there was a significant increase in patients with T1DM (from 56.7 (10.4) to 59.7 (13.2), *P* = 0.046). We observed no significant change in the insulin dose in patients with T1DM and T2DM. The frequency of FGM sensor use was 72% and 44% in patients with T1DM and T2DM, respectively.

Table 3 shows changes in scores of self-administered questionnaires before and after FGM initiation. No change was observed in DVS, IPAQ, and DTSQ scores in patients with T1DM. In contrast, a significant increase was seen in the number of patients engaging in moderate/high-level physical activity by IPAQ and in the treatment satisfaction score of DTSQ in patients with T2DM (from 26.0 (7.0) to 28.2 (6.6), *P* = 0.029). There were no significant changes in SDSCA score for patients with T2DM; however, foot care scores improved significantly in patients with T1DM.

## 4. Discussion

After the initiation of FGM, physical activity levels increased in patients with T2DM in our study. Yoo et al. [16] evaluated the effects of real-time CGM in patients with T2DM and found that exercise time per week became significantly longer. Allen et al. [26] reported that patients using a retrospective CGM significantly increased their number of steps per day. Although the CGMs used in these studies and FGM used in ours are different devices, the above results indicate that visually capturing a continuous blood glucose profile may improve a patient's awareness of exercise habits [27, 28]. In our study, FGM use significantly increased treatment satisfaction scores of DTSQ in patients with T2DM. Treatment satisfaction is closely associated with self-efficacy in patients with diabetes [29, 30]. FGM use enhances self-efficacy for exercise habits and treatment satisfaction, which may be linked to improvement in the glycemic profile. In our study, the exercise category scores for SDSCA were stable. Regarding the exercise category of SDSCA, the survey contents were scored based on two items: the number of days the patient participated in physical activity for at least 30 min and the number of days the patient performed exercises other than daily activities. In contrast, IPAQ is a reliable evaluation tool because it assesses both exercise time and intensity [22]. This may have enabled us to observe changes in physical activity not evaluated by SDSCA. In our study, there was no change in DVS before and after FGM use. Results similar to our findings have been reported by Yoo et al. [16]. They demonstrated no change in calorie intake and nutritional balance using real-time CGM. Cumulatively, these results demonstrated that FGM use provided favorable effects—increased physical activity—in patients with T2DM.

In the REPLACE study, a randomized controlled trial for patients with T2DM (mean age of 59 years and HbA1c of 8.6%) [14], FGM use led to decreased CV levels, an index of glycemic variability, and reduction of AAC of a glucose level of <70 mg/dL. However, there was no significant reduction in MAGE and HbA1c in the FGM group (intervention group), although the levels did tend to decrease. In contrast, blood glucose-related parameters, including MAGE and HbA1c, improved in patients with T2DM in our study. Several reasons may explain the differences in outcomes between the two studies. In the REPLACE study, the SMBG equipment that patients had been using was discontinued, and only FGM was used to measure their glucose level in the FGM group (intervention group). In contrast, patients concomitantly used both SMBG and FGM in our study. Glucose measurements with and without continuous SMBG may affect the results differently. Other reasons may include age, baseline HbA1c, and study design.

In the IMPACT study [13], which involved patients with T1DM (mean age of 42 years and HbA1c of 6.7%), FGM use had no significant effect on HbA1c; however, blood glucose-related parameters, including MAGE, did improve. Our results are similar to those of the IMPACT study, although the characteristics of the study groups were different, i.e., our study group had an older mean age and higher HbA1c values. However, there was no significant change in DVS, IPAQ, and SDSCA before and after FGM use in patients with T1DM in our study. At baseline, IPAQ and DVS were higher in patients with T1DM than in patients with T2DM in our study, i.e., changes in IPAQ or DVS might be smaller after FGM initiation. Further investigation is required to assess the effect of FGM use in patients with T1DM who have a sedentary lifestyle or consume poorly balanced foods.

Previous large-scale trials have shown that strict blood glucose control contributes to a decreased risk of vascular disease in patients with diabetes [3, 4]. In addition, a decrease in glycemic variability and improvement/maintenance of QOL are highly important. An earlier study reported that increased physical activity was a predictor of life prognosis of patients with diabetes, independent of improvements in the blood glucose profile [31]. To the best of our knowledge, this is the first study to demonstrate an increased score for physical activity on IPAQ, a reliable measurement tool, after the commencement of FGM use in patients with T2DM. Our study demonstrates the clinical benefit of FGM through higher physical activity in patients with T2DM.

This study had several limitations. First, the sample size was relatively small, possibly making the study underpowered. Second, the observation period was relatively short (12 weeks), and further studies are needed to evaluate FGM use over longer periods. Third, the DVS used to assess dietary variety did not evaluate calorie intake; decreased total calorie intake could play a role in the improvement in glucose-related parameters after the initiation of FGM. Finally, we did not include a control group in this study. Therefore, the results were insufficiently complete to support a causal relationship between FGM use and our findings. A study incorporating control groups will be needed to discuss such relationships further.

## 5. Conclusions

In this study, we investigated the effect of FGM use on dietary variety, physical activity, and self-care behavior in patients with diabetes. The results showed increased physical activity after using FGM in patients with T2DM. Thus, FGM was considered a useful tool to facilitate physical activity in these patients.

## Figures and Tables

**Table 1:** Characteristics of the diabetic study group.

Variables	Mean (SD) or %	Variables	Mean (SD) or %
Age (years)	57.3 (15.7)	Retinopathy, %	45.1
Women, %	52.0	Neuropathy, %	62.1
Body weight (kg)	64.1 (15.3)	Nephropathy, %	44.7
BMI (kg/m^2^)	24.3 (5.4)	CVD, %	14.2
T1DM/T2DM (numbers)	42/48	Medication	
HbA1c (%)	7.7 (1.2)	*α*-Glucosidase inhibitors, %	8.3
Duration of diabetes (years)	18.4 (11.2)	DPP4 inhibitors, %	37.5
Alcohol consumption, %	22.7	Sulfonylureas, %	16.6
Smoking, %	21.2	Biguanides, %	64.5
Hypertension, %	41.8	Glinides, %	4.1
Dyslipidemia, %	50.5	SGLT2 inhibitors, %	37.5
LDLC (mg/dL)	102.9 (27.7)	Pioglitazone, %	18.7
HDLC (mg/dL)	58.1 (18.5)	GLP-1 analog, %	15.5
TG (mg/dL)	144.2 (81)	Insulin, %	100

SD: standard deviation; BMI: body mass index; T1DM/T2DM: type 1 diabetes mellitus/type 2 diabetes mellitus; HbA1c: hemoglobin A1c; LDLC: low-density lipoprotein cholesterol; HDLC: high-density lipoprotein cholesterol; TG: triglyceride; GLP-1: glucagon-like peptide-1; DPP4: dipeptidyl peptidase 4; SGLT2: sodium-glucose cotransporter 2; CVD: cardiovascular diseases.

**Table 2:** Study parameters of the patients with type 1 and 2 diabetes before and after flash glucose monitoring intervention.

	T1DM (*n* = 42)			T2DM (*n* = 48)		
	Baseline	12 weeks	*P* value	Baseline	12 weeks	*P* value
HbA1c (%)	7.7 (1.3)	7.7 (1.2)	0.921	7.7 (1.2)	7.4 (0.8)	0.025^∗^
Body weight (kg)	56.7 (10.4)	59.7 (13.2)	0.046^∗^	71.5 (16.1)	71.2 (15.3)	0.607
SD (mg/dL)	71.2 (20.4)	66.2 (17.5)	0.033^∗^	53 (16.2)	45 (13.9)	<0.001^∗^
CV (%)	43.2 (9.3)	41.8 (9.3)	0.271	33.6 (8.3)	31.2 (9.4)	0.023^∗^
MAGE (mg/dL)	124.6 (31.9)	108.1 (28.4)	<0.001^∗^	93.3 (28.3)	81.3 (23.4)	0.001^∗^
MODD (mg/dL)	67.1 (18.7)	64.6 (19.6)	0.403	49.3 (20.7)	38.9 (16.3)	0.001^∗^
AAC of 70 mg/dL (mg/dL·h)	29.1 (34.2)	31.8 (51.4)	0.693	6.2 (10.1)	10.0 (23.2)	0316
AUC of 180 mg/dL (mg/dL·h)	577.3 (509.2)	509.4 (469.3)	0.2274	355.7 (555.4)	254.4 (501.4)	0.001^∗^
AUC of 180 mg/dL, 2 h after meals (mg/dL·h)						
Morning	63.1 (55.4)	53.2 (43.5)	0.125	43.3 (46.6)	46.1 (39.6)	0.936
Lunch	41.4 (27)	32.1 (27.1)	0.042^∗^	33.5 (23.6)	28.3 (22.6)	0.178
Dinner	33.2 (26.6)	35.6 (29.9)	0.519	27.9 (26.3)	22.1 (19.4)	0.104
Insulin dose (U/day)						
Bolas	23.3 (11.5)	21.5 (11.1)	0.071	13.1 (10.6)	13.0 (10.8)	0.915
Basal	15.4 (7.8)	13.6 (7.3)	0.105	15.1 (10.6)	14.7 (10.6)	0.207

Data are shown as mean (standard deviation). SD: standard deviation; MAGE: mean amplitude of glycemic excursions; CV: coefficient of variation; MODD: mean of daily difference; AAC: area above the curve; AUC: area under the curve; ^∗^*P* < 0.05.

**Table 3:** Study parameters of the patients with type 1 and 2 diabetes before and after flash glucose monitoring intervention.

	T1DM (*n* = 42)			T2DM (*n* = 48)		
	Baseline	12 weeks	*P* value	Baseline	12 weeks	*P* value
DVS (points), mean (SD)	3.2 (2.3)	3.2 (2.4)	0.899	2.1 (2.2)	2.3 (2.5)	0.655
IPAQ category, %			0.705			<0.001^∗^
Low	56.7	54.0		67.4	25.5	
Moderate/high	43.3	46.0		32.6	74.5	
SDSCA score (points), mean (SD)						
General diet	8.0 (4.4)	8.2 (3.7)	0.620	7.2 (3.1)	7.3 (2.9)	0.950
Specific diet	9.5 (3.3)	9.7 (3.2)	0.720	9.4 (4.9)	9.5 (3.8)	0.959
Exercise	5.3 (4.4)	4.9 (3.5)	0.493	5.3 (4.0)	5.4 (4.1)	0.917
SMBG	12.8 (3.0)	13.1 (2.2)	0.422	11.7 (3.6)	12.8 (1.9)	0.102
Drug	16.4 (4.1)	16.3 (3.8)	0.922	20.5 (1.3)	20.3 (1.7)	0.633
Foot care	21.7 (8.4)	25.5 (8.2)	0.002^∗^	21.7 (7.5)	24.1 (7.8)	0.058
DTSQ score (points), mean (SD)						
Perceived hyperglycemia	3.4 (1.5)	3.8 (1.4)	0.259	3.4 (1.4)	3.4 (1.6)	0.917
Perceived hypoglycemia	2.5 (1.4)	2.5 (1.3)	0.883	1.2 (1.4)	1.3 (1.3)	0.846
Treatment satisfaction	25.2 (6.5)	27.0 (5.8)	0.091	26.0 (7.0)	28.2 (6.6)	0.029^∗^

Data are shown as mean (standard deviation). DVS: Dietary Variety Score; IPAQ: International Physical Activity Questionnaire; SDSCA: Summary of Diabetes Self-Care Activities Measure; DTSQ: Diabetes Treatment Satisfaction Questionnaire; SMBG: self-monitoring of blood glucose.

## Data Availability

The data that support the findings of this study are available from the corresponding author upon reasonable request.
